# Structure and Properties of Epoxy Polysulfone Systems Modified with an Active Diluent

**DOI:** 10.3390/polym14235320

**Published:** 2022-12-05

**Authors:** Tuyara V. Petrova, Ilya V. Tretyakov, Alexey V. Kireynov, Alexey V. Shapagin, Nikita Yu. Budylin, Olga V. Alexeeva, Betal Z. Beshtoev, Vitaliy I. Solodilov, Gleb Yu. Yurkov, Alexander Al. Berlin

**Affiliations:** 1 N.N. Semenov Federal Research Center for Chemical Physics, Russian Academy of Sciences, 119991 Moscow, Russia; 2 Frumkin Institute of Physical Chemistry and Electrochemistry, Russian Academy of Sciences, 119071 Moscow, Russia; 3 N.M. Emanuel Institute of Biochemical Physics, Russian Academy of Sciences, 119334 Moscow, Russia

**Keywords:** epoxy oligomer, active diluent, polysulfone, physico-mechanical properties, crack resistance, matrix structure

## Abstract

An epoxy resin modified with polysulfone (PSU) and active diluent furfuryl glycidyl ether (FGE) was studied. Triethanolaminotitanate (TEAT) and iso-methyltetrahydrophthalic anhydride (iso-MTHPA) were used as curing agents. It is shown that during the curing of initially homogeneous mixtures, heterogeneous structures are formed. The type of these structures depends on the concentration of active diluent and the type of hardener. The physico-mechanical properties of the hybrid matrices are determined by the structure formed. The maximum resistance to a growing crack is provided by structures with a thermoplastic-enriched matrix-interpenetrating structures. The main mechanism for increasing the energy of crack propagation is associated with the implementation of microplasticity of extended phases enriched in polysulfone and their involvement in the fracture process.

## 1. Introduction

Currently, epoxy resins (EO) are widely used in many areas, such as matrices in high-performance composite materials, as well as the most common adhesives and sealants. Epoxy resins are widely used due to their good manufacturability and physical–mechanical properties that occur after their curing. This is especially important for plastics reinforced with high-strength fibers. Epoxy matrices, as a rule, have a rather low crack resistance. Typically, the G_IC_ energy value for epoxy matrices based on diglycidyl ether bisphenol A (DGEBA) is 0.1–0.4 kJ/m^2^ [1], and for carbon fiber-reinforced plastics is 0.3–0.5 kJ/m^2^ [2,3,4]. The crack resistance of matrices and reinforced plastics based on them can be increased by introducing modifiers of various nature: mineral or carbon particles [3,4,5,6,7,8,9,10], rubbers [11,12,13,14,15], active diluents, reactive copolymers [16,17,18,19], or thermoplastic modifiers [20,21,22,23,24,25,26,27,28,29]. The effect of introducing modifiers depends on their nature and structures, which are formed in the process of matrix formation.

The use of particles of different shapes and natures usually leads to an increase in physical–mechanical properties of about 70% [5,6,7,8,9,10]. The increase in crack resistance in this case is also associated with a growth in the crack path due to the rounding of the filler particles [30]. The highest values of fracture toughness of epoxy matrices can be achieved using rubbers as modifiers [11,12,13,14,15]. The effect of increasing the resistance to crack growth in the matrices is achieved due to the formation of a heterogeneous structure during the curing of the initial homogeneous mixture [11]. The energy of the growing crack is dissipated by elastic rubber particles. However, with this modification method, the heat resistance of matrices is noticeably reduced, which significantly narrows their application area. It is well known that the introduction of active diluents into epoxy oligomers does not lead to the formation of heterogeneous structures during their curing. The re-active modifier oligomers are incorporated into the polyepoxide network, thereby increasing its elasticity. This leads to a significant decrease in the glass transition temperature, and as a result, to a reduction in the use areas of such systems [16,17,18,19]. The most effective way to increase the crack resistance of epoxy matrices is the introduction of heat-resistant thermoplastics [20,21,22,23,24,25,26,27,28,29]. It should be taken into account that most rigid chain polymers are compatible with epoxy oligomers over a wide temperature range, i.e., dissolve in them. During the curing process, phase decomposition occurs with the formation of heterogeneous structures. These structures largely determine the resistance to crack growth due to the dissipation of its energy on a more plastic phase enriched with thermoplastic [29]. At the same time, the G_IC_ crack resistance values can approach 2 kJ/m^2^, namely, they increase by five times compared to unmodified systems [25,26,27,28,29]. It should be noted that the heat resistance (glass transition temperature) of modified systems does not decrease with this method of modification. However, in the process of the dissolution of the thermoplastic in the epoxy oligomer, the viscosity of the mixed compositions significantly increases [22,25,31,32,33,34], which greatly complicates their processing. For example, the viscosity of epoxy oligomers depending on temperature can be 0.01 to 18 Pa·s [22,31,34]. The introduction of thermoplastics into epoxy oligomers increases their viscosity. In the case of adding 20 wt. % polysulfone to EO, the value of the complex viscosity increases by 14 times at temperature 120 °C [22], for 25 wt. % polyetherimide in EO–by 120 times (from 0.07 to 8.50 Pa·s) at 120 °C [33], and for 20 wt. % polyethersulfone in EO–by 70 times (from 0.18 to 12.58 Pa·s) at 80 °C [34]. One way to reduce viscosity is to introduce active diluents into epoxy polymer systems. The addition of two modifiers to EO can lead to the formation of a heterogeneous system already at the stage of dissolution of the binder components. This will lead to the uncontrolled formation of the structure and the inability of high-quality processing of such a binder. Previously, we have shown that furan oligomers do not lead to phase decomposition of epoxypolsulfone blends at the mixing stage [35]. The introduction of the modifier, even in small amounts (10 wt. % of the epoxy oligomer), led to a noticeable decrease in the physico-mechanical properties of the epoxypolysulfone matrices, such as tensile strength, and impact toughness. At the same time, the viscosity could not be reduced to the values that are necessary to obtain low-defect reinforced fibrous composite materials by classical technologies [36].

Previously, we have determined the technological properties of an epoxy polysulfone binder modified with furfuryl glycidyl ether (FGE) [32]. Modification of epoxypolysulfone binders made it possible to reduce the processing temperature from 80 °C to 60 °C.

The viscosity of a polymer blend containing 38 wt. % polysulfone in EO with iso-methyltetrahydrophthalic anhydride hardener decreases from 20.5 Pa·s to ~3 Pa·s with the addition of 38 wt. % FGE at 60 °C. It made it possible to obtain low-viscosity epoxy polysulfone blends suitable to produce fiber-reinforced plastics by traditional methods (winding, contact pressing, vacuum forming, etc.). The compatibility of the active diluent with the epoxy polysulfone binder was evaluated visually during the dissolution of the components. As a result of the components mixing in the temperature range from 25 to 120 °C, the binder remained transparent. The temperature range of compatibility will allow the use of the developed binder in a wide range of technological parameters. In addition to the manufacture of reinforced plastics, the hybrid binder can be used as a compound with improved performance properties.

This work is an evolution of early studies [32] on the development of low-viscosity binders with increased crack resistance for fiber-reinforced composites. The purpose of this work is to study the effect of introducing an active diluent into epoxypolysulfone blends on their structure and physico-mechanical properties. In this work, complex studies of the physico-mechanical properties of polymer matrices, their structures were carried out, the mechanisms of destruction were determined, and the structures and properties of hybrid matrices were compared. The results of the study demonstrate the high efficiency of using hybrid polymer mixtures to obtain matrices with high crack resistance, strength, heat resistance, and good processability. Our earlier study [20] allows us to assert that the achieved properties of hybrid matrices are widely used in reinforced plastics for various applications.

## 2. Materials and Methods

### 2.1. Preparation of Polymer Mixtures

The epoxy oligomer (EO) was CHS EPOXY 520 resin (Spolchemie, Ustin nad Labem, Czech Republic), which was modified with active diluent furfuryl glycidyl ether (FGE (OOO “DOROS”, Yaroslavl, Russia)) and polysulfone (PSU) PSK-1 (AO “NIIPM”, Moscow, Russia) with a molecular weight of 35.000 g/mol.

To obtain the polymer blends, PSU was dissolved in EO at a temperature of 100–120 °C. The active diluent FGE was added to the resulting mixture at a temperature of 60–80 °C. The obtained polymer mixtures were cured with different types of hardeners: amine type triethanolamine titanate (TEAT (JSC CHIMEX Limited, St. Petersburg, Russia)) or anhydride type iso-methyltetrahydrophthalic anhydride (iso-MTHPA (JSC CHIMEX Limited, St. Petersburg, Russia)). The standard curing temperatures for the used hardeners may be different. A significant change in temperature and, as a consequence, the curing rate can lead to the formation of different types of phase structures in matrixes. The TEAT curing agent was introduced in an amount of 10 wt. % by weight EO + FGE, iso-MTHPA–90 wt. % (with a small stoichiometric excess). In the case of using iso-MTHPA, the accelerator 2-methylimidazole (2-MI (JSC CHIMEX Limited, St. Petersburg, Russia)) was added to the binder in the amount of 0.2 wt. % by weight of EO. The content of FGE and PSU in the epoxy binder ranged from 10 to 20 wt. %. by weight of EO for epoxyamine systems and 10–30 wt. % EO + iso-MTHPA.

### 2.2. Interferometry of Polymer Mixtures

The study of solubility and interdiffusion in binary (PSU–FGE, EO-FGE) systems was carried out using method of optical interferometry on an ODA-2 IPCE diffusiometer (IPCE RAS, Moscow, Russia) [37]. A helium–neon laser (λ = 632.8 nm) was used as a light source.

The method is based on the principle of in situ registration of optical density distribution in the interdiffusion zone of components and recording its change in time under the isobaric–isothermal conditions of the process [38]. The measurement method consisted in fixation of a PSU sample of 5 mm × 5 mm in size and about 150 µm-thick (obtained by pressing) or EO between the diffusion cell glasses, the inner surfaces of which are covered with a layer of translucent metal (Ni-Cr alloy) with a high reflection index. A small wedge angle of 2° was established between the glasses. After assembly, the cell was thermostated at a set temperature for at least 30 min. Then, the space between the glasses was filled with FGE.

All measurements were carried out in the temperature range from 20 to 160 °C. The experiments were carried out in the heating–cooling mode with a step of 5 °C and thermostated at each stage for at least 30 min.

**Figure 1:**
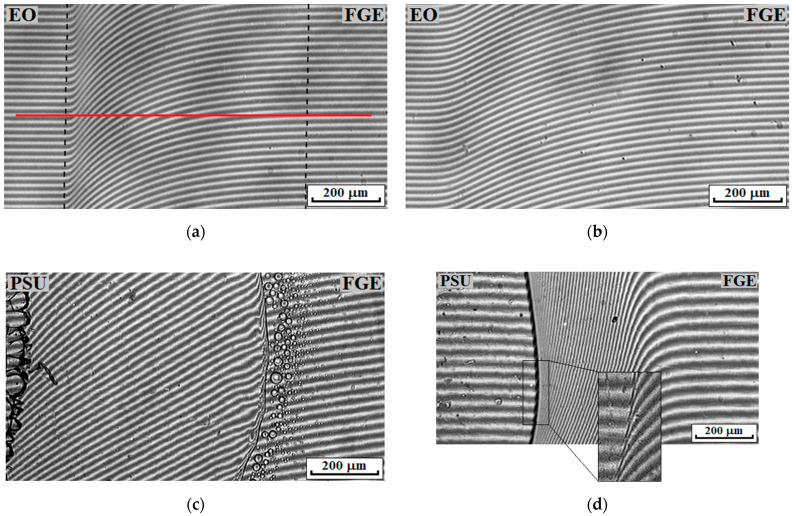
Interferograms of the interdiffusion zones of the EO–FGE (**a**,**b**) and PSU–FGE (**c**,**d**) systems at 20 (**a**,**c**) and 160 °C (**b**,**d**).

The methods for processing the interferograms of the interdiffusion zones and constructing phase diagrams (PDs) did not differ from those described earlier [39,40]. In the interdiffusion zone (between the dashed lines) a solid line parallel to the interference fringes in the region of pure components is drawn (Figure 1a). The ratio C = 1/N, where N is the number of intersections of the solid line with the interference fringes in the interdiffusion zone, equals the concentration increment when passing from one interference fringe to another. The compositions of coexisting phases were calculated using the formula Ci = C*Ni, where Ni is the number of crossings of the solid line with interference fringes to the left (or right) of the phase boundary or heterogeneous zone.

### 2.3. Differential Scanning Calorimetry

The method of differential scanning calorimetry (DSC) (NETZSCH DSC 204 F1 Phoenix, NETZSCH-Gerätebau GmbH, Selb, Germany) was used to determine the thermal effects of the curing process of modified binders and the glass transition temperature of matrices in the measurement temperature range of 25–250 °C, with a heating rate of 10 K/min^−1^ [41].

### 2.4. Physico-Mechanical Studies

Samples for physical and mechanical studies were obtained by free molding. Before foundry, the prepared compositions were degassed in a vacuum oven at a temperature of 60–70 °C for 15–20 min. After that, they were poured into silicone molds and cured according to the following conditions: 8 h/160 °C when added to the TEAT polymer mixture; 2 h/90 °C, 14 h/120 °C–iso-MTHPA. After curing, the matrices were mechanically processed, and then tested for tension, splitting (crack resistance) and dynamic mechanical analysis. 

Tensile testing of the modified epoxy matrices was carried out on a Zwick Z010 universal testing machine (ZwickRoell GmbH & Co., Ulm, Germany) on blade-shaped specimens [24]. The samples were loaded at a constant rate of 1 mm/min. Tensile diagrams were used to determine strength σ, elastic modulus E, and elongation ε at fracture.

The crack resistance of G_IR_ matrices was determined by splitting a double-cantilever beam (I-section) [20] on an Instron 3365 universal testing machine (Instron, Norwood, MA, USA). The initial notch on an I-beam for crack initiation was applied with a blade 0.2–0.3 mm-thick at a distance of 8–10 mm from the edge of the sample. The specific fracture toughness for each crack growth was determined by the following Formula (1) [20].

G_IR_ = 2 × γ_F_ = P_i_ × δ_i_ × k/4 × l_i_ × w_i_ (1)

where γ_F_ is the specific surface energy of crack growth; P_i_ is the maximum force at which the i-th crack propagation begins; δ_i_ = Li/M–compliance (deflection of the ends of the sample at the points of application of the load); l_i_ is the distance from the center of the holes for fixing the sample to the end of the i-th crack; w_i_ is the average width of the surface during the growth of the i-th crack; k–is the experimental constant of the sample, depending on its rigidity (k = 3).

The morphology of the fracture surface after splitting the samples was examined using a Phenom ProX scanning electron microscope (Thermo Fisher Scientific, Waltham, MA, USA). 

The method of dynamic mechanical analysis (DMA) was used to determine the temperature dependences of the modulus of elasticity (E′–T) and the tangent of the mechanical loss angle (tg δ–T) on flat rectangular samples using a three-point loading scheme on a DMA 242 E Artemis Netzsch device (NETZSCH-Gerätebau GmbH, Selb, Germany). The glass transition temperatures of the phases were determined from the peaks of the tg δ–T curve. The size of the samples was 20 mm × 6 mm × 2 mm. The temperature was changed from 25 to 250 °C at a heating rate of 2 K/min. The load application frequency is 1 Hz.

## 3. Results and Discussion

### 3.1. Compatibility of Polymer Blend Components

The compatibility of the three components in the investigated systems (PSU, EO, and FGE) was studied by analyzing bicomponent mixtures (PSU–EO, PSU–FGE, and EO–FGE) over the full range of concentrations and temperatures (from 25 up to 160 °C).

Previously, in [42], it was shown that the PSU–EO system is completely compatible over the full concentration temperature range from 25 to 270 °C. Figure 1 shows typical interferograms characterizing the solubility of the components of the PSU–FGE and EO–FGE systems at different temperatures. 

The continuous change in isoconcentration bands (refractive index) in the diffusion zones for all compositions indicates that the EO–FGE system is fully compatible over the full temperature process range. In the case of the PSU–FGE system, complete compatibility of the components is observed at temperatures above 33 °C. Lowering the temperature leads not only to the vitrification of polysulfone-concentrated solutions, but also to the appearance of a phase boundary in the region of dilute solutions, separating the zone of dissolution of the mixture components among each other in accordance with the Flory–Huggins theory of polymer solutions [39,43]. The data obtained on phase equilibria in bicomponent systems indicate the complete compatibility of the studied components at the stage of mixture preparation in the range from 25 to 160 °C. Thus, a homogeneous mixture is formed upon mutual dissolution of the components of ternary systems at 100 °C. Further addition of a curing agent to the system and temperature activation of the process of forming a three-dimensional spatial network of chemical bonds will lead to an increase in viscosity, a decrease in thermodynamic compatibility, and, as a result, the formation of phase structures [42].

### 3.2. Differential Scanning Calorimetry (DSC) of Polymer Blends

Thermograms of the curing reaction of the modified hybrid resines were obtained by DSC metod. The temperatures of the beginning T_onset_, maximum T_peak_, and end T_end_ of the curing reactions and their enthalpy were determined from the analysis of exothermic peaks. Table 1 shows that the shift of the exothermic peak is insignificant with a change in the content of modifiers for both the TEAT-based resine and iso-MTHPA. However, there is a “broadening” of the exothermic peak for compositions with a concentration of 15 and 20 wt. % PSU based on TEAT at a content of 20 wt. % FGE, which confirms the increase in the temperature of the end of the curing reaction up to ~190 °C. Apparently, this is due to the influence of the active thinner, which reduces the rate of the curing reaction. For resins based on iso-MTHPA, the concentration of modifiers has little effect on the exothermic peak of the curing of the resins. For mixtures containing iso-MTHPA or TEAT, the enthalpy of the curing reaction decreases with the addition of modifiers.

### 3.3. Dynamic Mechanical Analysis (DMA)

**Figure 2:**
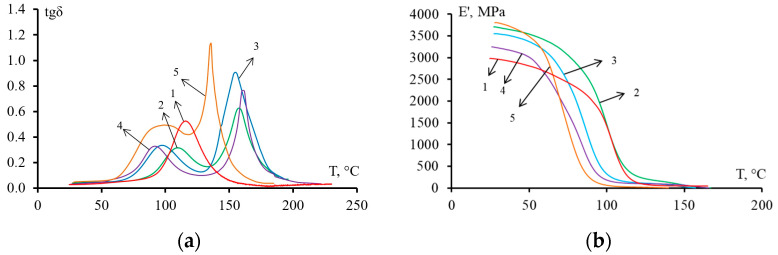
Mechanical loss tangent angle tan δ (**a**) and elastic modulus E′ (**b**) of modified epoxy matrices cured with TEAT versus temperature T. The ratio of modifiers PSU/FGE: 1—P0/F0; 2—P20/F0; 3—P20/F10; 4—P20/F20; 5—P15/F20.

**Figure 3:**
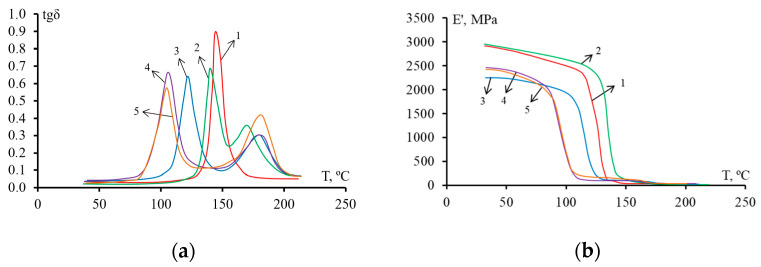
Mechanical loss tangent angle tan δ (**a**) and elastic modulus E′ (**b**) of modified epoxy matrices cured with iso-MTHPA versus temperature T. The ratio of modifiers PSU/FGE: 1—P0/ F0; 2—P20/F0; 3—P20/F10; 4—P20/F20; 5—P30/F20.

The dependence of tg δ and E′ on temperature for epoxy polysulfone matrices modified with active diluent and cured with TEAT or iso-MTHPA are shown in Figure 2 and Figure 3, respectively. On the curves tg δ corresponding to the unmodified epoxy matrix, one peak is observed. However, for the modified systems, two peaks are already observed on the curves. This behavior indicates the heterogeneous structure of the matrix, which was formed during the curing of the hybrid resine. The first peak belongs to the “thermosetting phase”, the second–to the “thermoplastic phase”. The maxima on the curves tan δ–T corresponds to the glass transition temperature of each of the phases.

The dependence curves E′–T (Figure 2b and Figure 3b) show that for mixed compositions cured with iso-MTHPA or TEAT, the addition of FGE reduces the glass transition temperature, and as a result, the phase transition shifts to lower temperatures (inflections of curves). Matrices based on compositions containing an active diluent have a lower elastic modulus compared to an epoxy polysulfone matrix. Such a decrease in the elastic modulus occurs due to the plasticization of the epoxy matrix with the addition of an active diluent.

The glass transition temperatures T_g_ of the matrices determined by DMA and DSC are shown in Table 2.

According to the DMA data, it can be concluded that the addition of FGE and PSU reduces the glass transition temperature of the “thermosetting phase” of hybrid epoxy matrices. Introduction 20 wt. % PSU in the epoxy matrix reduces T_g_ by 5 °C for the system cured with TEAT, by 4 °C for the cured iso-MTHPA. An increase in the content of the FGE active diluent in epoxypolysulfone matrices reduces the T_g_ values of the thermoset-enriched phase. At a concentration of 20 wt. % FGE in the system EO + 20 wt. % PSU, the T_g_ values of the “thermosetting phase” decrease by 18 °C for TEAT-based matrices, by 35 °C for iso-MTHPA. The decrease in the glass transition temperature of the thermoset-enriched phase is apparently associated with the plasticizing effect of the FGE active diluent. For the studied compositions, the glass transition temperatures of the “thermoplastic phase” practically do not differ from each other. The exceptions are systems cured with TEAT, with a content of PSU–15 wt. % and 20 wt. % FGE. For this system, the T_g_ value decreased by 22 °C compared to the EO + 20 wt. % PSU. For the EO + 20 wt. % PSU + iso-MTHPA system, a lower glass transition temperature (164 °C) is observed. According to DSC data, the glass transition temperatures of epoxy matrices based on both curing agents change by only 6–9 °C with the addition of 20 wt. % PSU. The content of FGE slightly (no more than 4 °C) reduces the glass transition temperature of epoxy polysulfone matrices cured with TEAT. For epoxypolysulfone matrices containing FGE and iso-MTHPA, the plasticizing effect is more pronounced: the glass transition temperature decreases by approximately 20 °C. 

### 3.4. Mechanical Properties of Hybrid Matrices

**Figure 4:**
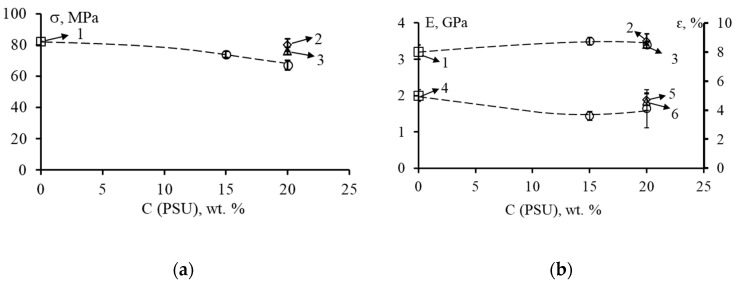
Strength σ, elastic modulus E and relative elongation ԑ at tension of epoxy systems EO + 20 wt. % of FGE, cured by TEAT, depending on the content of FGE: (**a**) 1—P0/F0, 2—P20/F0, 3—P20/F10; (**b**) 1 and 4—P0/F0, 2 and 5—P20/F0, 3 and 6—P20/F10. In (**b**) the upper curve corresponds to the modulus of elasticity, the lower curve corresponds to the relative elongation.

**Figure 5:**
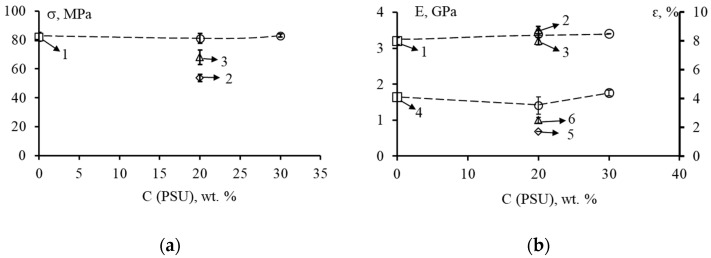
Strength σ, modulus of elasticity E and relative elongation ԑ at tension of epoxy systems EO + 20 wt. % FGE, cured iso-MTHPA, depending on the content of FGE: (**a**) 1—P0 /F0, 2—P20/F0, 3—P20/F10; (**b**) 1 and 4—P0/F0, 2 and 5—P20/F0, 3 and 6—P20/F10. In (**b**) the upper curve corresponds to the modulus of elasticity, the lower curve corresponds to the relative elongation.

The values of strength σ, elastic modulus E and relative elongation ԑ of unmodified and modified matrices are shown in Figure 4 and Figure 5. 

The tensile strength of the unmodified matrix (EO + TEAT) is 82 MPa (Figure 4a, point 1). When 20 wt. % PSU is introduced into this system (Figure 4a, point 2), the tensile strength of the epoxy-polysulfone matrix remains virtually unchanged (80 MPa). The addition of 20 wt. % FGE to this EO + PSU system leads to a slight (up to 16%) decrease in the values of strength σ. In this case, the higher the concentration of PSU, the greater the effect of reducing the strength σ is observed with the introduction of an active diluent (Figure 4a, dashed line). The elastic modulus E of the modified matrices (Figure 4b) remains almost unchanged both when PSU is added and when FGE is added to the epoxy oligomer. The E values are in the range of 3.2–3.5 GPa and are typical of most epoxy matrices.

At a content of 20 wt. % PSU in EO, the relative elongation εp differs little from the values of the elongation of the unmodified matrix (Figure 4b, point 5). The addition of 20 wt. % FGE in the EO + PSU composition reduces the values of ԑp to 4.1%. With a decrease in the content of PSU to 15 wt. % in the system EO + 20 wt. % FGE, the value of ԑ decreases to 3.6%. Thus, the introduction of an active diluent into epoxy polysulfone matrices somewhat reduces their deformability. 

For modified epoxy matrices cured with iso-MTHPA, a different effect is observed when an active diluent is added to them (Figure 5). The complex modification of the PSU epoxy matrix and 20 wt. % FGE (Figure 5a, dashed line) practically does not change the values of strength σ (~80 MPa), relative elongation (~4%), and elastic modulus (~3.4 GPa).

It should be noted that the introduction of 20 wt. % PSU into EO slightly reduces the strength (up to 54 MPa) and elongation at fracture (up to 1.7%) (Figure 5 currents 2 and 5). Slightly higher values of σ and ε are characteristic of EO + 10 wt. % FGE + 20 wt. % PSA matrices. In this case, σ = 68 MPa, ε = 2.5% (Figure 5 points 3 and 6). 

### 3.5. Crack Resistance

**Figure 6:**
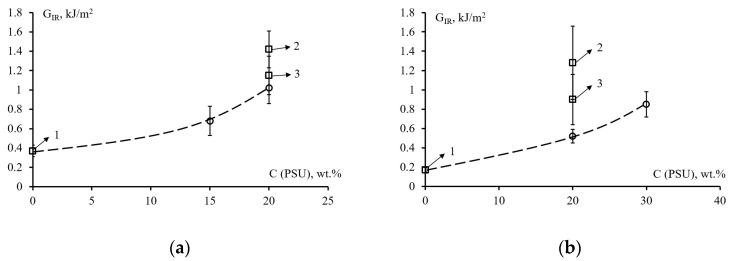
Crack resistance G_IR_ of epoxy systems EO + 20 wt. % FGE, cured with TEAT (**a**) or iso-MTHPA (**b**), modified with thermoplastic modifier PSU: 1—P0/F0; 2—P20/F0; 3—P20/F10.

The values of crack resistance of systems EO + 20 wt. % FGE versus the concentration of PSU for epoxy matrices with different contents of FGE and PSU, cured with TEAT or iso-MTHPA are shown in Figure 6. Point 1 on the graphs corresponds to unmodified epoxy matrices. Thus, for EO cured TEAT, the crack resistance is 0.37 kJ/m^2^, and for cured iso-MTHPA it is 0.17 kJ/m^2^. The modification of EO with polysulfone (20 wt. %) leads to a noticeable increase in the crack resistance of such matrices. The G_IR_ values for the TEAT-cured epoxy-polysulfone matrix are 1.42 kJ/m^2^, for iso-MTHPA cured 1.28 kJ/m^2^ (point 2 in Figure 6). 

The effect of polysulfone on the crack resistance of the epoxy matrix modified with 20 wt. % FGE is shown by the dashed line in Figure 6. In this case, polysulfone also significantly increases the crack resistance of matrices. For the matrix composition EO + 20 wt. % FGE + 20 wt. % PSU + TEAT, the G_IR_ value is 1.02 kJ/m^2^, EO + 20 wt. % FGE + 30 wt. % PSU + iso-MTHPA is 0.85 kJ /m^2^. Reducing the FGE content to 10 wt. % in the epoxy matrix with 20 wt. % PSU increases the crack resistance value to 1.15 and 0.9 kJ/m^2^ depending on the type of curing agent (point 3). Thus, it can be concluded that the introduction of polysulfone into epoxy matrices significantly increases their crack resistance.

Additional modification of epoxy polysulfone matrices with an active diluent to reduce the viscosity of hybrid binders leads to some decrease in the resistance of matrices to crack growth by 28–59%, depending on the type of curing agent. However, compared with unmodified matrices, the G_IR_ values for matrices of the composition EO + 20 wt. % FGE + 20 wt. % PSU are ~3 times higher. A significant increase in crack resistance with the addition of a thermoplastic is primarily associated with phase decomposition during the curing of the binder. The decrease in resistance to crack growth with the addition of an active diluent can be explained by the type of matrix microstructure formed after curing, which are given below.

### 3.6. Morphology of Hybrid Epoxypolysulfone Matrices

**Figure 7:**
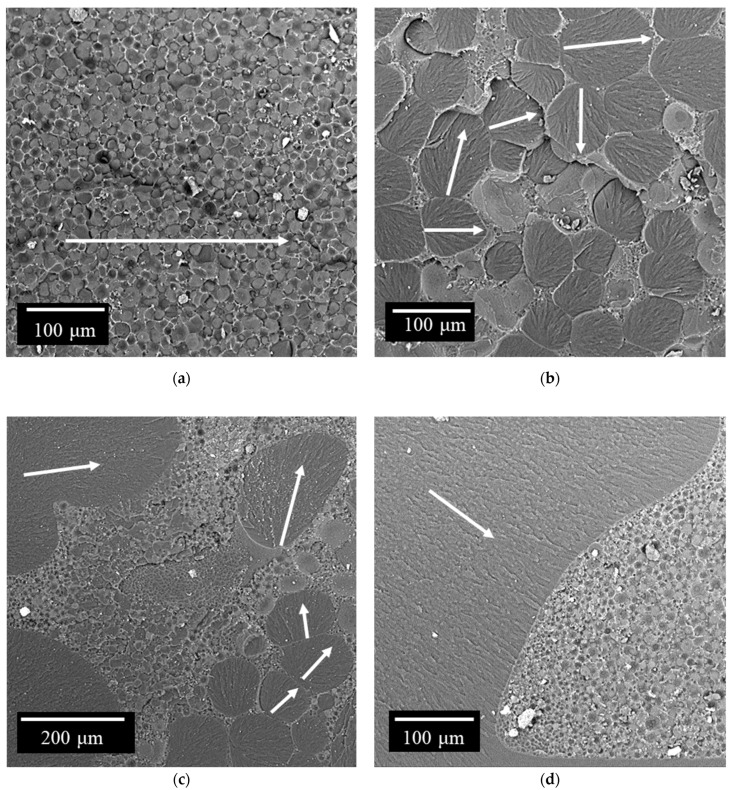
Micrographs of the surface of cracks in the epoxy matrix cured with TEAT and modified PSU/FGE (wt. %): (**a**) 20/0; (**b**) 20/10; (**c**) 20/20; (**d**) 15/20.

**Figure 8:**
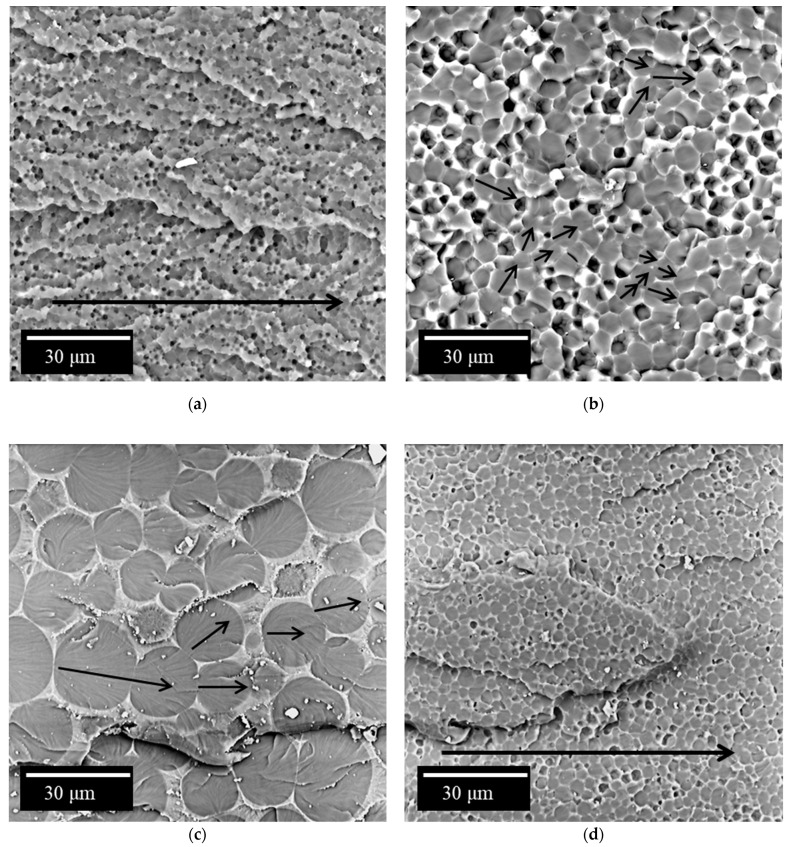
Micrographs of the surface of cracks in the epoxy matrix cured with iso-MTHPA and modified with PSU/FGE (wt. %): (**a**) 20/0; (**b**) 20/10; (**c**) 20/20; (**d**) 30/20.

The surface morphology of cracks in epoxy polysulfone matrices modified with an active diluent is shown in Figure 7 and Figure 8. It can be seen from the micrographs that the cured modified systems represent a heterogeneous structure. The type of structure formed depends on the amount of polysulfone, active diluent and type of hardener in the epoxy matrix. The mechanism of phase decomposition for such systems was studied by us earlier in [42]. 

Changing the concentration of polysulfone and curing temperature affect the formation of not only the structure of matrices dispersion, but also more complex structures. Depending on the content of thermoplastics and the type of hardener, the matrix can be enriched with epoxy oligomer (at concentrations less than 10 wt. %) or enriched with thermoplastic (thermoplastic concentration of about 20 wt. %). It should be noted that the maximum effect of increasing the crack resistance of epoxy polymer matrices was observed at high concentrations of the thermoplastic modifier when the matrix was enriched with thermoplastic [20,24,34,40]. The influence of the active diluent on the phase structure of hybrid matrices with a high (20 wt. %) content of polysulfone and on the mechanisms of crack formation will be considered below.

#### 3.6.1. Polymer Blends Cured with TEAT

The addition of 20 wt. % PSU to the epoxy matrix leads to the formation of a “matrix-dispersion” type structure in the cured composition. The matrix in such a system is enriched with polysulfone, which confirms the presence of the characteristic Ka line of sulfur in the EDX spectrum. The dispersed phase is epoxy oligomer. The size of the dispersed phase varies from 3 to 20 µm (Figure 7a). The continuous phase in this case represents thin interlayers between dispersed particles. The introduction of an active FGE diluent into this system leads to a change in the size distribution of particles and a change in the type of organization of the phase structure. The addition of 10 wt. % FGE to 20 wt. % PSU leads to an increase in the size of the dispersed phase enriched in epoxide. This behavior of the system is due, on the one hand, to a decrease in the viscosity of the system, and, as a consequence, to an increase in the interdiffusion coefficients of the components. On the other hand, when adding FGE, the relative Upper Critical Solution Temperature (UCST) does not decrease in comparison with the bicomponent system (PSU–EO), but rather increases, since for the PSU–FGE system, UCST corresponds to 33 °C. It is known that the curing reaction leads to an expansion of the heterogeneous region of the phase diagram and an intersection of the process isotherm. Thus, the phase decomposition will occur at lower degrees of conversion (α). As a result, and because of the better mobility of the components in the initial low-viscosity system, the formation of phase structures will begin at higher diffusion coefficients compared to the system without FGE. Under these conditions, large (33–133 µm) phases enriched in EO are formed in the cured composition (Figure 7b).

**Figure 9:**
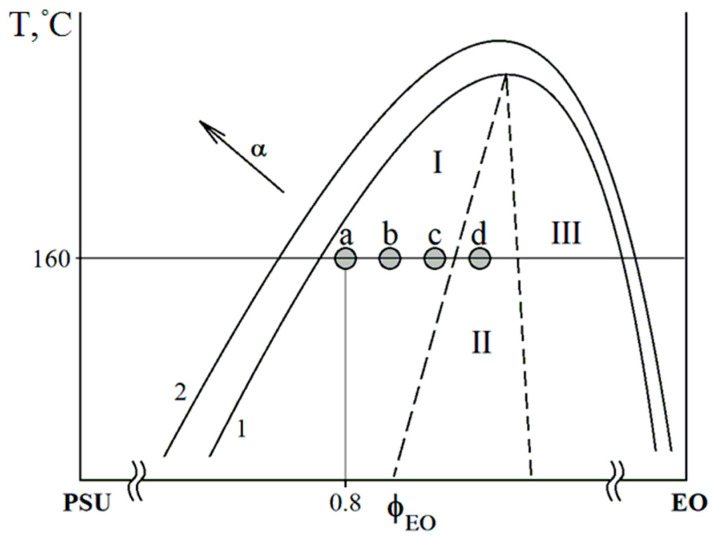
Schematic representation of PD in progress curing, where: 1—PD model systems PSU–EO; 2—model PD PSU–EO + FGE. o–figurative points of the curable systems PSU/FGE (wt. %): a—20/0, b—20/10, c—20/20, d—15/0. I, III—PD regions, where a “matrix-dispersion”; II—PD region, where a structure of the “interpenetrating phases” type is formed.

It should be noted that the volume fraction of the continuous phase enriched with PSU in the EO–PSU system decreases with the addition of FGE. This is due to the shift of the figurative point of the cured system to the region of solutions diluted in terms of the thermoplastic and the expansion of the heterogeneous region of the PD of the initial components (Figure 9). In the continuous phase of the polysulfone, an EO-enriched dispersed phase is formed with a particle size of 0.6–3.8 µm. Additionally, in the phase enriched with epoxy oligomer, a dispersed polysulfone phase with a size of 0.9–2.2 μm is formed. The formation of such a complex multilevel phase structure occurs when the figurative point of the cured system enters the labile region of the PD, where the phase decomposition proceeds according to the spinodal mechanism. Phases are formed at a high mobility of the components due to the onset of phase decomposition at a low degree of conversion.

Increasing the concentration of FGE to 20 wt. % in the epoxy-polysulfone mixture further reduces the viscosity of the system, expands the binodal curve PD, and shifts the figurative point of the curable mixture, bringing it closer to the PD region corresponding to the “interpenetrating phase” structure. Such changes, presented in the temperature-concentration field of the PD (Figure 9), lead to structural changes shown in Figure 7c, where the size of the dispersed EO phase in the extended PSU phase is 2–7 μm, and the size of the dispersed phase of PSU in EO–1.2–2.4 μm. A decrease in the PSU concentration to 15 wt. % in the hybrid matrix leads to the formation of an “interpenetrating phase” structure (Figure 7d).

#### 3.6.2. Iso-MTHPA Cured Polymer Blends

As with the TEAT-cured polymer systems, the addition of 20 wt. % PSU to the iso-MTHPA-cured epoxy matrix results in the formation of a “matrix-dispersion” structure. In this case, too, the matrix is enriched in polysulfone, and the dispersed phase is enriched in epoxy oligomer. The size of the dispersed phase varies from 0.8 to 3 µm (Figure 8a). The difference from the curable TEAT system is the lower curing temperature and the much higher concentration of curing agent. In connection with the last difference, a comparative analysis of the sizes of phase structures is impossible. From a qualitative analysis point of view, the systems behaved similarly. The introduction of FGE led to a shift of the figurative point of the curable system in the temperature-concentration field to the right to the region of solutions diluted with PSU and the expansion of the heterogeneous region of PD, which was reflected in an increase in the volume fraction of the polysulfone matrix. A decrease in the viscosity of the mixture of the initial mixture and the onset of phase decomposition at lower degrees of conversion and high diffusion constants led to an increase in the size of disperse structures enriched in epoxide. For the hybrid matrix EO + 20 wt. % PSU + 10 wt. % FGE, the size of the EO-enriched phase is 0.9–8.2 µm (Figure 8b); for EO + 20 wt. % PSU +20 wt. % FGE is a dispersed phase enriched with EO with dimensions of 3.7–24 µm (Figure 8c). 

An increase in the concentration of PSU to 30 wt. % at a content of 20 wt. % FGE led to the opposite effect, namely, to a shift of the figurative point to the left and, as a result, a reduction in the volume fraction of the thermoplastic matrix and a decrease in the size of dispersed structures (up to 0.4–6 μm) Apparently, this is the result of an increase in viscosity due to an increase in the concentration of PSU and the onset of phase decomposition at higher degrees of conversion and, as a consequence, low diffusion coefficients.

### 3.7. Mechanisms for Increasing the Strength of Hybrid Matrices

As noted above and in [20,42], a significant increase in crack resistance (from 3.8 to 7.5 times depending on the type of curing agent) is observed when a large amount (20 wt. %) of polysulfone is introduced into the epoxy matrix. During the destruction of unmodified epoxypolysulfone matrices, an increase in the crack propagation energy can be associated with the realization of the microplasticity of the matrix enriched with polysulfone. A growing crack expends a significant amount of energy to deform and fracture the more plastic continuous phase (Figure 7a and Figure 8a). Presumably, the dispersed phase of EO makes a small contribution to the dissipation of the crack energy. In the case of the formation of the PSU structure, the dispersion of EO with the content of the FGE active diluent in such a matrix (Figure 8b,d) crack resistance values are somewhat lower (0.85–0.9 kJ/m^2^). Apparently, the active diluent helps to reduce the micro deformability of the PSU-enriched phase and embrittlement of the EO-enriched phase. For matrices with such structure, no explicit correlation between the crack propagation energy and the sizes of EO-enriched phases is observed. In this case, the fracture toughness values are in the range from 0.85 to 1.42 kJ/m^2^. The crack resistance of matrices, the structure of which is formed by primary and secondary phase decomposition (Figure 7b,c and Figure 8c), is somewhat lower than for composites with the structure of the PSU matrix–EO dispersion. For such structures, depending on the type of curing agent and concentration, the crack resistance values vary from 0.52 to 1.15 kJ/m^2^. The decrease in the crack propagation energy can be associated with a decrease in the volume occupied by the PSU-enriched phase. Thus, the PSU phase is less involved in the process of crack formation. Furthermore, it can be seen on microphotographs that the extended phases of PSU can change the direction of microcrack growth, thereby increasing the total energy consumption for macrocrack propagation. 

With crack growth in matrices having a structure with interpenetrating phases (Figure 7d), the contribution of the extended thermoplastic phase is lower than for structures in which the volume of the PSU phase is larger. In this case, the crack resistance of such systems is 0.68 kJ/m^2^.

Summarizing the above, it can be argued that the higher the involvement of the extended phase enriched in polysulfone (the larger the volume it occupies), the higher the resistance to crack growth. At the same time, the maximum effect of increasing crack resistance can be achieved when forming a system with the structure of the PSU matrix–EO dispersion. Reducing the share of such structures by modifying the FGE or changing the temperature–time conditions for the formation of compositions leads to a decrease in the crack resistance of materials. It should be noted that heterogeneous structures with extended thermoplastic phases almost always have significantly greater resistance to the formation and propagation of cracks.

## 4. Conclusions

The study of the solubility of the bicomponent systems PSU–FGE and EO–FGE showed that the complete compatibility of the components at the stage of mixture preparation occurs in the temperature range from 25 to 160 °C. Through the mutual dissolution of the components of ternary systems at 100 °C, a homogeneous mixture is formed. The subsequent addition of a curing agent to the system will lead to an increase in viscosity, a decrease in thermodynamic compatibility and, therefore, the formation of phase structures. The presence of phase structures formed while curing was confirmed by DMA studies of hybrid matrices. Two peaks were found on the dependences tg δ–T, which correspond to the phase enriched with EO and the phase enriched with thermoplastic. It is shown that the glass transition temperature of the phase enriched with PSU remains virtually unchanged with varying concentrations of modifiers. Such a change in the glass transition temperature is primarily associated with the plasticizing effect from the introduction of an active diluent into the system. The effect of matrix plasticization is also confirmed by the downward trend in the glass transition temperature of hybrid matrices by DSC method. 

The introduction of polysulfone (20 wt. %) into epoxy matrices significantly increases their crack resistance from 4 to 7 times, depending on the type of curing agent. Additional modification of epoxy polysulfone matrices with an active diluent to reduce the viscosity of hybrid binders leads to a decrease in the resistance of matrices to crack growth by 28–59% relative to EO + 20 wt. % PSU systems, depending on the type of curing agent. However, compared with unmodified matrices, the G_IR_ values for matrices of the composition EO + 20 wt. % FGE + 20 wt. % PSU are ~3 times higher. 

The maximum effect of increasing crack resistance can be achieved by forming a system with the structure of the PSU matrix–EO dispersion. Reducing the proportion of such structures by modifying the FGE or changing the temperature–time conditions for the formation of compositions leads to a decrease in the crack resistance of materials.

Heterogeneous structures with extended thermoplastic phases are almost always much more resistant to the formation and propagation of cracks. At the same time, it can be considered that the main contribution to the increase in the crack resistance of hybrid matrices is made by the mechanism for implementing the microdeformability of extended thermoplastic phases—it is not possible to obtain a significant increase in the values of ε for modified systems with the introduction of FGE and PSU.

The active diluent in this case can be considered as a component that gives an additional degree of freedom in controlling the final phase structure of the cured epoxy polysulfone systems. This allows it to be used to create heterogeneous matrices with a given phase structure with a wide range of structural parameters.

## Figures and Tables

**Table 1:** Thermal effect ΔH and temperatures of the onset T_onset_, peak T_peak_, and end T_end_ of the curing reaction of the modified epoxy resin.

Content of PSU, wt. %	Content of FGE, wt. %	T_onset_, °C	T_peak_, °C	T_end_, °C	ΔH, J/g	T_onset_, °C	T_peak_, °C	T_end_, °C	ΔH, J/g
		TEAT				Iso-MTHPA			
0	0	139	160	171	37.7	136	175	207	365.8
20	0	135	164	177	27.4	143	176	204	280.1
20	10	128	157	169	32.7	144	175	204	297.7
20	20	134	165	189	32.4	144	176	207	269.9
15	20	133	165	190	32.3	-	-	-	-
30	20	-	-	-	-	137	175	206	238.1

**Table 2:** Glass transition T_g_ temperatures of epoxy matrices modified with PSU and FGE obtained by DMA and DSC methods.

Sample	T_g_, °C					
	DMA				DSC	
	Thermosetting Phase		Thermoplastic Phase			
	TEAT	Iso-MTHPA	TEAT	Iso-MTHPA	TEAT	iso-MTHPA
P0/F0	115	145	-	-	98	133
P20/F0	110	141	157	164	107	139
P20/F10	97	123	153	181	106	112
P20/F20	92	106	160	178	103	111
P15/F20	99	-	135	-	108	-
P30/F20	-	104	-	180	-	105

## Data Availability

Data presented in this study are available on request from the corresponding author.
